# Compound heterozygous *LPIN2* pathogenic variants in a patient with Majeed syndrome with recurrent fever and severe neutropenia: case report

**DOI:** 10.1186/s12881-019-0919-3

**Published:** 2019-11-14

**Authors:** Jun Liu, Xu-Yun Hu, Zhi-Peng Zhao, Ruo-Lan Guo, Jun Guo, Wei Li, Chan-Juan Hao, Bao-Ping Xu

**Affiliations:** 1 China National Clinical Research Center of Respiratory Diseases, Respiratory Department of Beijing Children’s Hospital, Capital Medical University, National Center for Children’s Health, Beijing, 100045 China; 2 Beijing Key Laboratory for Genetics of Birth Defects, Beijing Pediatric Research Institute; MOE Key Laboratory of Major Diseases in Children; Genetics and Birth Defects Control Center, Beijing Children’s Hospital, Capital Medical University, National Center for Children’s Health, Beijing, 100045 China; 3 Henan Key Laboratory of Pediatric Inherited & Metabolic Diseases, Henan Children’s Hospital, Zhengzhou Hospital of Beijing Children’s Hospital, Zhengzhou, 450018 China

**Keywords:** Majeed syndrome, Fever, Neutropenia, Autosomal recessive

## Abstract

**Background:**

Majeed syndrome is a rare, autosomal recessive autoinflammatory disorder first described in 1989. The syndrome starts during infancy with recurrent relapses of osteomyelitis typically associated with fever, congenital dyserythropoietic anemia (CDA), and often neutrophilic dermatosis. Mutations in the *LPIN2* gene located on the short arm of chromosome 18 have been identified as being responsible for Majeed syndrome.

**Case presentation:**

We report an 8-month-old boy, who presented with recurrent fever, mild to moderate anemia, and severe neutropenia. Erythrocyte sedimentation rate and C-reactive protein were elevated. Molecular testing identified a paternal splicing donor site variant c.2327 + 1G > C and a maternal frameshift variant c.1691_1694delGAGA (Arg564Lysfs*3) in *LPIN2.*

**Conclusions:**

Only a few cases with *LPIN2* mutation have been reported, mainly in the Middle East with homozygous variants. Our patient exhibited a mild clinical phenotype and severe neutropenia, different from previous reports.

## Background

Majeed syndrome is a rare, autosomal recessive autoinflammatory disorder first described in 1989. The syndrome starts during infancy with recurrent relapses of osteomyelitis typically associated with fever, congenital dyserythropoietic anemia (CDA), and often neutrophilic dermatosis. Mutations in the *LPIN2* gene, located on the short arm of chromosome 18, have been identified as being responsible for Majeed syndrome. Here we report what we believe to be the first case of Majeed syndrome in a Chinese individual. This case is of variable severity.

## Case presentation

### Clinical information

This Chinese 8-month-old boy presented at the age of 6 months with recurrent fever lasting for 5–7 days, recurring every 3–7 days. Sometimes he had a slight cough. He had no physical pain or movement problems. He had no rash or other symptoms. The infant was born full term. The delivery was normal delivery with a birth weight of 3.0 kg. His parents had a non-consanguineous marriage. There was a neonatal history of jaundice. The boy had mild pallor when he was admitted to our hospital. He had no lymphadenopathy or hepatosplenomegaly. Blood routine examination showed severe neutropenia (380–400/mm^3^) with normal white blood cell count, microcytic anemia (hemoglobin 85–95 g/L), and slight thrombocytosis. The boy had an elevated erythrocyte sedimentation rate (79 mm/h) and C-reactive protein (39 mg/L, normal< 8 mg/L). Immunoglobulin and lymphocyte subsets were found to be normal. Rheumatoid factors were negative. Antinuclear antibody was positive with a titer of 1:80, while the anti-ds DNA antibody was negative. Antineutrophil cytoplasmic antibodies showed mild elevated anti-MPO antibody (30.2 RU/ml) and negative anti-PR3 antibody. Thyroid function was normal. Serum iron and transferrin levels were low, which indicated iron-deficiency anemia. Bone marrow hemocytology revealed myleoproliferation cells, and the proportion of myelocyte was decreased because of the granulocytosis. Red blood cells were active and tended to proliferate. Metarubricytes dominated with small, hollow, and distorted mature erythrocytes. Blood and bone marrow puncture specimens were cultured for bacteria and fungi and showed no growth. Results of viral serologic studies were also negative. Lymphocyte interferon release assay was negative. Abdominal ultrasound scan gave normal findings. Cardiac ultrasound showed no abnormalities. Chest CT showed no interstitial or parenchymal infiltration. Because he had no limbs pain, the patient did not undergo an MRI scan.

### Molecular genetic studies

After obtaining informed consent, we isolated DNA from peripheral blood samples obtained from the patient and parents using the Gentra Puregene Blood Kit (Qiagen, Hilden, Germany). Whole exome library was captured by a SureSelect Human All Exon Kit (Agilent Technologies, Santa Clara, CA, USA) according to the manufacturer’s instructions. Target regions were sequenced and aligned to the GRCh37/hg19 human reference sequence. Variants were annotated and filtered by TGex (tgex-app.genecards.cn). Variants were classified following the ACMG/AMP standards and guidelines [1]. Putative pathogenic variants were confirmed by Sanger sequencing.

## Discussion and conclusions

**Fig. 1:**
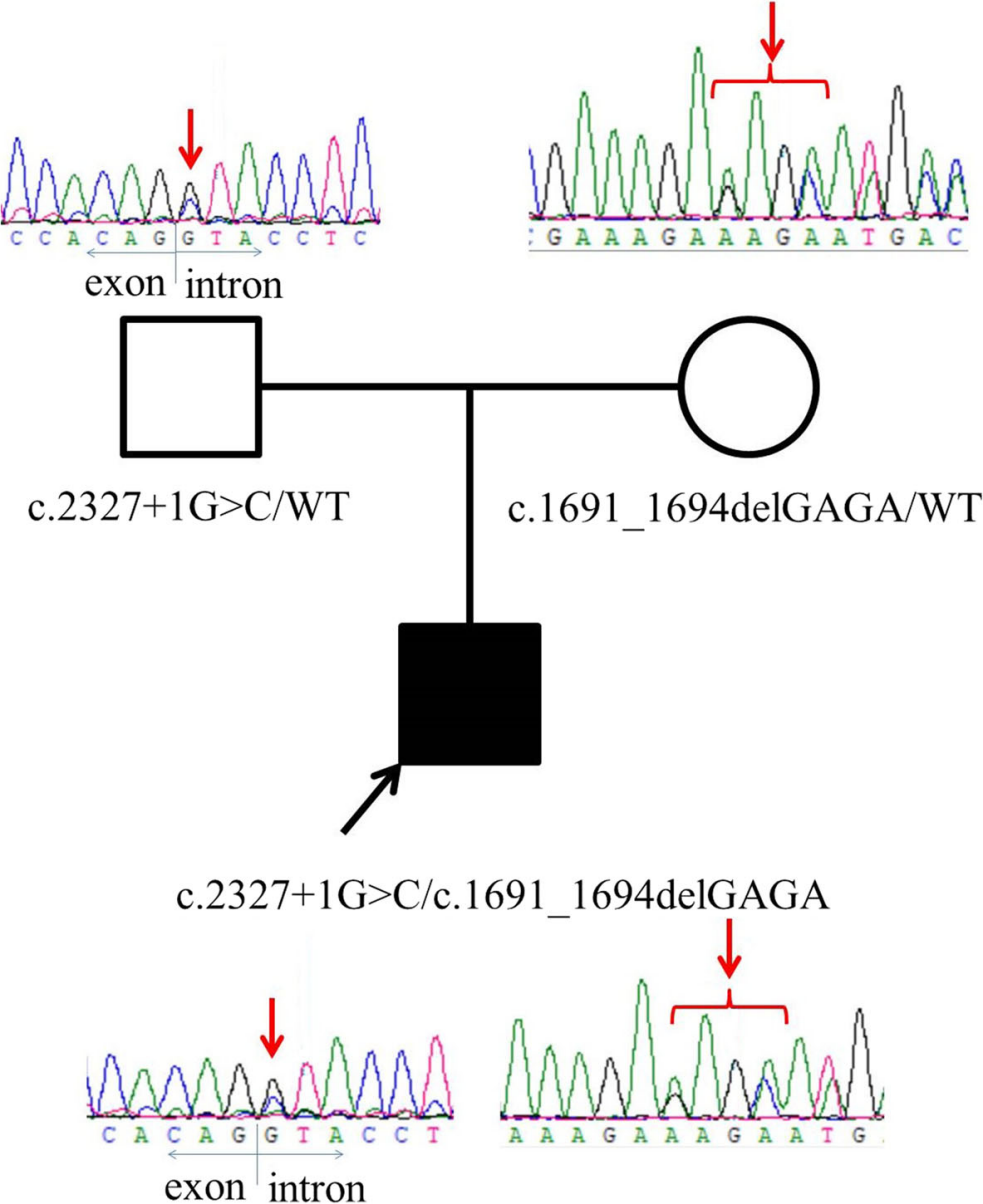
Pedigrees of *PLIN2* mutation family and Sanger sequencing

After sequencing, we identified a paternal splicing donor site variant c.2327 + 1G > C and a maternal frameshift variant c.1691_1694delGAGA (Arg564Lysfs*3) in *LPIN2* (NM_014646.2, Fig. 1). c.2327 + 1G > C has not been reported in dbSNP, 1000 genome, ESP, ExAC, or gnomAD databases, indicating it is very rare in normal populations. This variant was predicted to disarrange the donor site according to Human Splicing Finder (www.umd.be/HSF3/HSF.shtml) and caused exon 17 deletion or intron 17 insertion either entirely or partly. This variant was first reported in an Arabic family. The proband was a 3-year-old girl with Majeed syndrome [2]. The author predicted this variant could produce an R776S change followed by 65 amino acids prior to encountering a stop codon in intron 17. c.1691_1694delGAGA, located in exon 12, led to premature termination codon at position 3 amino acids after mutation. It is expected to produce a truncated protein or lead to early degradation of mRNA through the mechanism of nonsense-mediated decay. This variant has not been reported in dbSNP, ESP, or 1000 genome databases. The frequency in ExAC database was 0.000008236, suggesting that the frequency was extremely low. Both variants were classified as pathogenic variants according to ACMG/AMP guidelines.

Majeed syndrome is a rare autosomal recessive disorder characterized by chronic recurrent multifocal osteomyelitis (CRMO). This is an early-onset disorder with a lifelong course and congenital dyserythropoietic anemia (CDA) that presents as hypochromic, microcytic anemia during the first year of life and ranges from mild to severe enough to render the patient transfusion dependent. Some individuals also develop a transient inflammatory dermatosis, often manifesting as Sweet syndrome (neutrophilic skin infiltration). It is often accompanied by recurrent fever. The diagnosis is based on clinical findings and molecular genetic testing of *LPIN2*, the only gene in which pathogenic variants are known to cause Majeed syndrome. Only a few cases with *LPIN2* mutation have been reported, mainly in the Middle East with homozygous variants [2–9].

**Fig. 2:**
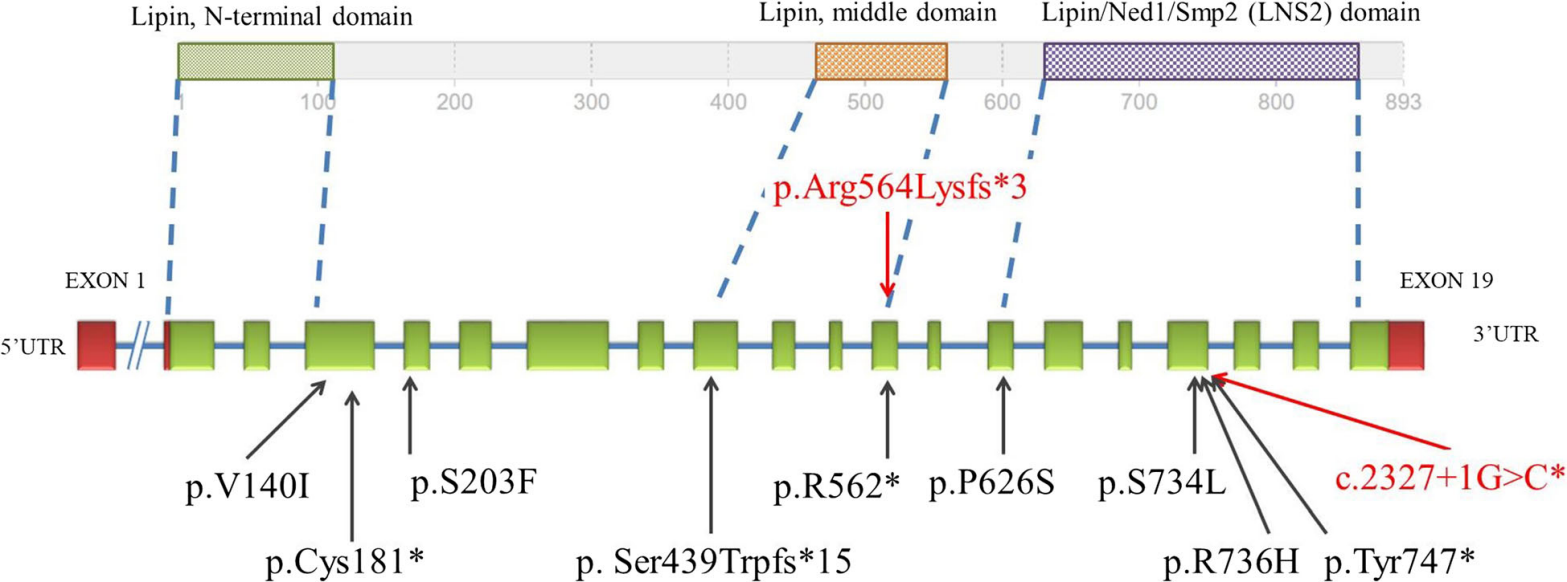
Distribution of variants in exonic location of *LPIN2* and domain structure of the Lipin2 protein The structure of the protein is shown in the upper row with crucial domains, drawn approximately to scale. The structure of the *LPIN2* is shown in the lower row. Two structures are linked by a dashed line to indicate exonic locations of respective domains. Variants above (red) are reported in this study. Variants shown in black below are previously reported in the literature. c.2327 + 1G > C has already been reported by Al-Mosawi (2007)

*LPIN2* encodes a phosphatidate phosphatase that plays important roles in controlling the metabolism of fatty acids at different levels. The function of LPIN2 is not well known. According to a previous study, it acts as a magnesium-dependent phosphatase, converting phosphatidic acid to diacylglycerol in the biosynthesis of triglycerides, phosphatidylcholine, and phosphatidylethanolamine. It can also act as a nuclear transcriptional coactivator of PPARGC1A and so regulate lipid metabolism [10, 11]. Homozygous knock out *Lpin2* mice displayed increases in mean platelet volume, red blood cell distribution, and lymph nodes and decreases in mean corpuscular hemoglobin, bone mineral density, and overall bone mass. They also had abnormal circulating phosphate level, hydrometra, and preweaning lethality with incomplete penetrance. *LPIN2* has 19 exons and 3 lipin domains located in N-terminal (amino acid: 1–108), middle (amino acid: 469–561), and C-terminal (amino acid: 677–831), respectively (Fig. 2). Lipin domains are highly conserved in lipin proteins and lipin homologues from *Saccharomyces cerevisiae* (Smp2, PAH1) and Schizosaccharomyces pombe (Ned1) and the function of these domains was still unclear. Mutations on lipin domains in mice lead to fatty liver dystrophy.

We here report the first case of Majeed syndrome in the individual of Chinese heritage and with variable severity. Our patient exhibited a mild clinical phenotype, unlike in previously reported cases (Table 1). He had recurrent fever and mild to moderate hypochromic and microcytic anemia without severe CRMO. He had no physical pain, swelling, or movement disorders. Majeed reported a Palestinian Arab boy who presented at the age of 2 months with recurrent episodes of high fever and irritability [12]. At the age of 9 months, these episodes began to be associated with periarticular swellings with heat, tenderness, and limitation of movement. Therefore, the patient’s signs and symptoms need to be observed continuously. Our patient had severe neutropenia from the age of 6 months, and his absolute neutrophil count was 380–400/mm^3^. This phenotype has been reported in few cases. Mosawi reported an Arabic female with Majeed syndrome who had mild neutropenia (1080/mm^3^) in the neonatal period [3]. RAO reported a 15-year-old boy with Majeed syndrome complicated by mild neutropenia [5]. Those cases suggest that neutropenia may be part of the phenotype. More cases must be studied to confirm this phenotype. Treatment with IL-1 blockade was reported in Majeed syndrome [13]. Our patient did not use IL-1 blockade because of his age and the mildness of his phenotype.

**Table 1:** Fourteen patients with Majeed syndrome: clinical course and laboratory investigations

author	Gender	Age at onset/diagnosis	Clinical features	Laboratory tests	Genetic testing	others
Mosawi [2]	F	10 m/3 y	CRMO	ESR 66–96 mm/h CRP 30 mg/L Neutropenia (750/mm^3^) Hb 7.5–9.5 g/dL	c.2327 + 1G > C	Fever Dyserythropoietic anemia Neutropenia
Ferguson [3]	F	–	CRMO	–	c.2201C > T p.s734 L	Mild fever Microcytosis Sweet syndrome Relative with psoriasis
	M	–	CRMO	–	c.2201C > T p.s734 L	Mild fever Microcytosis Sweet syndrome Relative with psoriasis
	M	–	CRMO	–	c.2201C > T p.s734 L	Mild fever Microcytosis Relative with psoriasis
	M	–	CRMO	–	c.2201C > T p.s734 L	Mild fever Microcytosis Sweet syndrome Relative with psoriasis
	–	–	CRMO	–	c.540_541delAT p.(Cys181*)	High fever Microcytosis Frequent blood transfusion pustulosis
	–	–	CRMO	–	c.540_541delAT p.(Cys181*)	High fever Microcytosis Frequent blood transfusion
Herlin [4]	M	6 m/29 m	CRMO	ESR 92 mm/h CRP 19.6 mg/L Hb 9.7 g/dL	c.1316_1317delCT P.(Ser439Trpfs*15)	Dyserythropoietic anaemia
	M	3 m/13 m	CRMO	ESR 96 mm/h CRP 23.7 mg/L Hb 9.0 g/dL	c.1316_1317delCT P.(Ser439Trpfs*15)	Dyserythropoietic anemia Fever
Rao [5]	M	2 y/15 y	CRMO	ESR 53–140 mm/h Hb 7.9–9.9 g/dL Neutropenia (2840–4230/mm^3^)	c.2241_2243delinsGG	Mild neutropenia Dyserythropoietic Anemia
	M	8 y/13 y	Milder CRMO	ESR 45 mm/h Hb 11.2 g/dL	c.2241_2243delinsGG	Mild anemia
Moussa [6]	M	6 m/5y	CRMO	ESR14-92 mm/h CRP < 5-14 mg/L Hb9.7–12.1 g/dL	c.2327 + 1G > C p.s734 L	No skin lesions Mild anemia
	F	4 y/14 y	CRMO	ESR20–68 mm/h CRP < 5–59 mg/L Declining Hb to 8 g/dL at age 15 years	c.2327 + 1G > C p.s734 L	No skin lesions Idiopathic scoliosis Rheumatic fever Dyserythropoietic anemia
Roy [13]	M	–	CRMO	High inflammatory	c.2207 G > A p.R736H	Microcytic anaemia

Majeed syndrome is an autosomal recessive, autoinflammatory disorder. It is characterized by CRMO and CDA. Our patient had the compound heterozygous LPIN2 pathogenic variant and exhibited a mild clinical phenotype, unlike in previously reported cases.

## Data Availability

The datasets during and/or analyzed during the current study are available from the corresponding author on reasonable request.

## Abbreviations

CDA: Congenital dyserythropoietic anemia
CRMO: Chronic recurrent multifocal osteomyelitis
CRP: C-Reactive protein

## References

[1] Richards S, Aziz N, Bale S, Bick D, Das S, Gastier-Foster J, Grody WW, Hegde M, Lyon E, Spector E (2015). Standards and guidelines for the interpretation of sequence variants: a joint consensus recommendation of the American College of Medical Genetics and Genomics and the Association for Molecular Pathology. Genet Med.

[2] Al-Mosawi ZS, Al-Saad KK, Ijadi-Maghsoodi R, El-Shanti HI, Ferguson PJ (2007). A splice site mutation confirms the role of LPIN2 in Majeed syndrome. Arthritis Rheum.

[3] Ferguson PJ, Chen S, Tayeh MK, Ochoa L, Leal SM, Pelet A, Munnich A, Lyonnet S, Majeed HA, El-Shanti H (2005). Homozygous mutations in LPIN2 are responsible for the syndrome of chronic recurrent multifocal osteomyelitis and congenital dyserythropoietic anaemia (Majeed syndrome). J Med Genet.

[4] Herlin T, Fiirgaard B, Bjerre M, Kerndrup G, Hasle H, Bing X, Ferguson PJ (2013). Efficacy of anti-IL-1 treatment in Majeed syndrome. Ann Rheum Dis.

[5] Rao AP, Gopalakrishna DB, Bing X, Ferguson PJ (2016). Phenotypic variability in Majeed syndrome. J Rheumatol.

[6] Monies D, Abouelhoda M, AlSayed M, Alhassnan Z, Alotaibi M, Kayyali H, Al-Owain M, Shah A, Rahbeeni Z, Al-Muhaizea MA (2017). The landscape of genetic diseases in Saudi Arabia based on the first 1000 diagnostic panels and exomes. Hum Genet.

[7] Moussa T, Bhat V, Kini V, Fathalla BM (2017). Clinical and genetic association, radiological findings and response to biological therapy in seven children from Qatar with non-bacterial osteomyelitis. Int J Rheum Dis.

[8] Omoyinmi E, Standing A, Keylock A, Price-Kuehne F, Melo GS, Rowczenio D, Nanthapisal S, Cullup T, Nyanhete R, Ashton E (2017). Clinical impact of a targeted next-generation sequencing gene panel for autoinflammation and vasculitis. PLoS One.

[9] Marzano AV, Ortega-Loayza AG, Ceccherini I, Cugno M (2018). LPIN2 gene mutation in a patient with overlapping neutrophilic disease (pyoderma gangrenosum and aseptic abscess syndrome). JAAD Case Rep.

[10] Donkor J, Zhang P, Wong S, O'Loughlin L, Dewald J, Kok BP, Brindley DN, Reue K (2009). A conserved serine residue is required for the phosphatidate phosphatase activity but not the transcriptional coactivator functions of lipin-1 and lipin-2. J Biol Chem.

[11] Gropler MC, Harris TE, Hall AM, Wolins NE, Gross RW, Han X, Chen Z, Finck BN (2009). Lipin 2 is a liver-enriched phosphatidate phosphohydrolase enzyme that is dynamically regulated by fasting and obesity in mice. J Biol Chem.

[12] Majeed HA, Al-Tarawna M, El-Shanti H, Kamel B, Al-Khalaileh F (2001). The syndrome of chronic recurrent multifocal osteomyelitis and congenital dyserythropoietic anaemia. Report of a new family and a review. Eur J Pediatr.

[13] Roy NBA, Zaal AI, Hall G, Wilkinson N, Proven M, McGowan S, Hipkiss R, Buckle V, Kavirayani A, Babbs C. Majeed syndrome: description of a novel mutation and therapeutic response to bisphosphonates and IL-1 blockade with anakinra. Rheumatology (Oxford). 2019:1–3. 10.1093/rheumatology/kez317 PMC7571481 31377798

